# Bis(tetra­ethyl­ammonium) bis­(dimethyl­formamide)­tetra­kis­(μ-*N*,2-dioxido­benzene-1-carboximidato)penta­copper(II)

**DOI:** 10.1107/S1600536811007975

**Published:** 2011-03-12

**Authors:** Jacob Herring, Matthias Zeller, Curtis M. Zaleski

**Affiliations:** a Department of Chemistry, Shippensburg University, 1871 Old Main Drive, Shippensburg, PA 17257, USA; b Department of Chemistry, Youngstown State University, One University Plaza, Youngstown, OH 44555, USA

## Abstract

The title compound, (C_8_H_20_N)_2_[Cu_5_(C_7_H_4_NO_3_)_4_(C_3_H_7_NO)_2_], abbreviated as (TEA)_2_[Cu^II^(12-MC_Cu^II^_ _N(shi)_-4](DMF)_2_ [where TEA is tetra­ethyl­ammonium, shi^3−^ is salicyl­hydroximate (or *N*,2-dioxidobenzene-1-carboximidate) and DMF is *N*,*N*-dimethyl­formamide], contains five Cu^II^ ions. Four of the Cu^II^ ions are members of a metallacrown ring (MC), while the fifth Cu^II^ is bound in a central cavity. Two of the ring Cu^II^ ions are five-coordinate with distorted square-pyramidal geometry. The coordination sphere is composed of two shi^3−^ ligands and one DMF mol­ecule. The other two ring Cu^II^ ions and the central Cu^II^ ion are four-coordinate with square-planar geometry. The coordination spheres of these ions are only composed of shi^3−^ ligands. The charge of the [Cu^II^(12-MC_Cu^II^_ _N(shi)_-4]^2−^ unit is balanced by two uncoordinated TEA^+^ countercations. The structure shows severe static disorder with the metallacrown, the tetra­ethyl­ammonium cations and the DMF solvent mol­ecule all disordered over each of two mutually exclusive sites, with occupancy rates for the major moieties of 0.6215 (6) for the metallacrown, 0.759 (3) for the tetra­ethyl­ammonium ion and 0.537 (6) for the DMF mol­ecules. The metallacrown unit is located on a crystallographic inversion center and disordered about a non-crystallographic twofold axis. The DMF mol­ecule and the tetra­ethyl­ammonium ion are disordered about a non-crystallographic twofold axis and pseudo-inversion center, respectively.

## Related literature

For a general review of metallacrowns, see: Mezei *et al.* (2007 ▶); Pecoraro (1989 ▶); Pecoraro *et al.* (1997 ▶). For related [Cu(12-MC_Cu^II^_ _N(ligand)_-4)]^2−^ structures, see: Gibney *et al.* (1994 ▶). For structure analysis of a two-dimensional chiral solid based on a Cu^II^[12-MC_Cu^II^_-4)]^2+^ complex, see: Bodwin & Pecoraro (2000 ▶). For single-crystal X-ray structure analysis of related Mn^II^(OAc)_2_[12-MC_Mn^III^_ _N(shi)_-4], where ^−^OAc is acetate, see: Lah *et al.* (1989 ▶). For an explanation on how to calculate τ, see: Addison *et al.* (1984 ▶). 
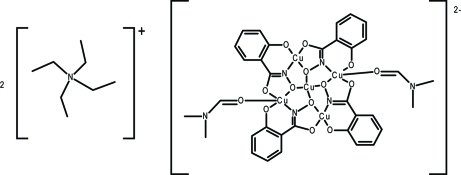


## Experimental

### 

#### Crystal data

(C_8_H_20_N)_2_[Cu_5_(C_7_H_4_NO_3_)_4_(C_3_H_7_NO)_2_]
*M* *_r_* = 1325.74
Orthorhombic, 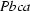

*a* = 16.641 (3) Å
*b* = 13.616 (2) Å
*c* = 23.238 (4) Å
*V* = 5265.4 (15) Å^3^
*Z* = 4
Mo *K*α radiation
μ = 2.06 mm^−1^
*T* = 100 K
0.45 × 0.40 × 0.29 mm

#### Data collection

Bruker SMART APEX CCD diffractometer
Absorption correction: multi-scan (*APEX2*; Bruker, 2009 ▶) *T*_min_ = 0.588, *T*_max_ = 0.746
51635 measured reflections
8316 independent reflections
6387 reflections with *I* > 2σ(*I*)
*R*_int_ = 0.055

#### Refinement

*R*[*F* ^2^ > 2σ(*F* ^2^)] = 0.043
*wR*(*F* ^2^) = 0.104
*S* = 1.12
8316 reflections
631 parameters
101 restraints
H-atom parameters constrained
Δρ_max_ = 0.47 e Å^−3^
Δρ_min_ = −0.35 e Å^−3^

### 

Data collection: *APEX2* (Bruker, 2009 ▶); cell refinement: *APEX2*; data reduction: *APEX2*; program(s) used to solve structure: *SHELXS97* (Sheldrick, 2008 ▶); program(s) used to refine structure: *SHELXL97* (Sheldrick, 2008 ▶); molecular graphics: *SHELXTL* (Sheldrick, 2008 ▶), *Mercury* Macrae *et al.* (2006 ▶) and *Ortep-3 for Windows* (Farrugia, 1997 ▶); software used to prepare material for publication: *SHELXL97*.

## Supplementary Material

Crystal structure: contains datablocks I, global. DOI: 10.1107/S1600536811007975/jj2076sup1.cif

Structure factors: contains datablocks I. DOI: 10.1107/S1600536811007975/jj2076Isup2.hkl

Additional supplementary materials:  crystallographic information; 3D view; checkCIF report

## Figures and Tables

**Fig. 1.**
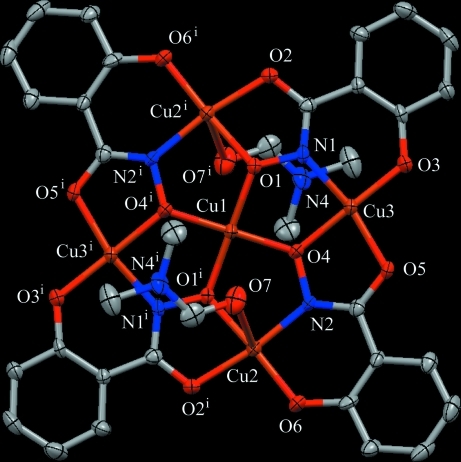
Single-crystal X-ray structure (top view) of (TEA)2[Cu(12—MCCuIIN(shi)-4)](DMF)2 (1). The thermal ellipsoid plot of 1 is at a 50% probability level with the disordered portions of the molecule shown only at the higher occupancy positions. All non-carbon atoms are labeled. Hydrogen atoms and the lattice TEA have been omitted for clarity (symmetry operator (i): -x + 1, -y + 1, -z + 1). Color scheme for all figures: orange - CuII, red - oxygen, blue - nitrogen, and gray - carbon.

**Fig. 2.**
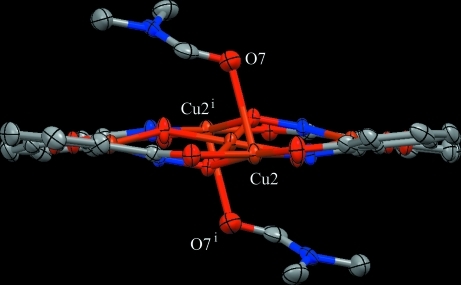
Single-crystal X-ray structure (side view) of 1. The DMF is coordinated to Cu2 with a CuII—O7 distance of 2.763 (14) Å. The thermal ellipsoid plot of 1 is at a 50% probability level with the disordered portions of the molecule shown only at the higher occupancy positions. Cu2 and O7 are labeled to highlight the DMF molecules bonded to the metallacrown. Hydrogen atoms and the lattice TEA have been omitted for clarity (symmetry operator (i): -x + 1, -y + 1, -z + 1).

**Fig. 3.**
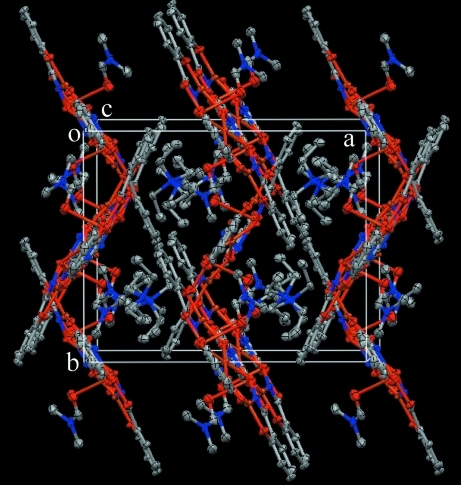
Packing diagram of 1 along the c axis. The thermal ellipsoid plot of 1 is at a 50% probability level with the disordered portions of the molecule shown only at the higher occupancy positions. Hydrogen atoms have been omitted for clarity.

**Fig. 4.**
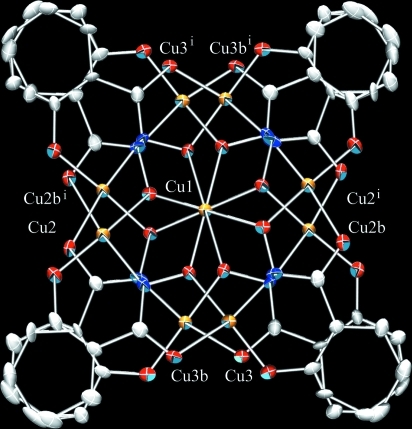
Single-crystal X-ray structure (top view) of 1. The thermal ellipsoid plot of 1 is at a 50% probability level. All disordered atoms of the MC are shown. The metallacrown is disordered over two mutually exclusive sites by a non-crystallographic twofold axis. The refined occupancy ratio is 0.6215 (6) to 0.3785 (6). All copper atoms are labeled. Hydrogen atoms, the DMF molecules, and the lattice TEA have been omitted for clarity (symmetry operator (i): -x + 1, -y + 1, -z + 1).

**Fig. 5.**
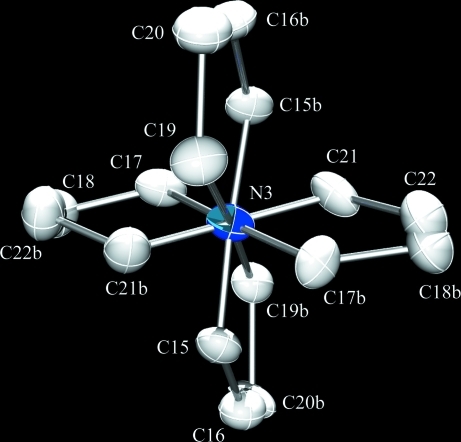
Single-crystal X-ray structure of the TEA countercation with all disordered atoms shown. The thermal ellipsoid plot is at a 50% probability level. The tetraethylammonium is disordered over two mutually exclusive sites by a pseudo-inversion center. The refined occupancy ratio is 0.759 (3) to 0.241 (1). Hydrogen atoms have been omitted for clarity.

**Fig. 6.**
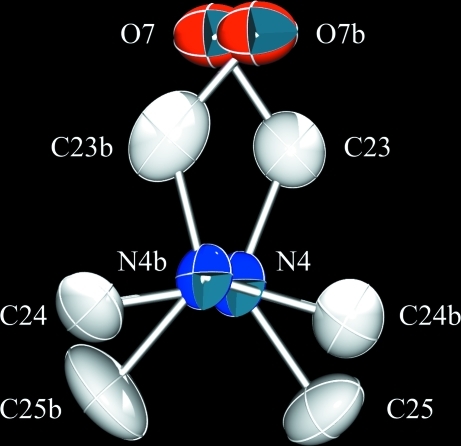
Single-crystal X-ray structure of the DMF molecule with all disordered atoms shown. The thermal ellipsoid plot is at a 50% probability level. The DMF is disordered over two mutually exclusive sites by a non-crystallographic twofold axis. The refined occupancy ratio is 0.537 (6) to 0.463 (6). Hydrogen atoms have been omitted for clarity.

## supplementary crystallographic information

### Comment

Since the identification of metallacrowns (MC) in 1989 (Pecoraro, 1989), these inorganic crown ether analogues have proved to be very diverse molecules (Mezei *et al.*, 2007; Pecoraro *et al.*, 1997). Metallacrowns can behave as single-molecule magnets, have potential use as MRI contrast agents, and can selectively bind cations or anions (Mezei *et al.*, 2007). In addition to being inorganic structural and functional analogues of crown ethers, the naming scheme for the two molecules is very similar. For example, the name 12-MC-4 indicates that there are 12 atoms in the metallacrown ring and there are 4 oxygen atoms in the ring that can potentially bind to a central metal ion. A complete nomenclature description for metallacrowns can be found in Pecoraro *et al.* (1997).

Copper(II) 12-MC-4 structures are common (Mezei *et al.*, 2007), and the structures tend to be fairly planar. The planar structures are generated by placing the ring Cu^II^ ions at 90^o^ relative to each other. This placement is typically achieved by selection of a ligand, such as salicylhydroxamic acid, that can form fused five- and six-membered chelate rings. However, planar structures have been observed for other sized fused chelate rings (Mezei *et al.*, 2007). One planar Cu^II^[12-MC_Cu^II^_-4]^2+^ has been used to build a two-dimensional chiral solid (Bodwin & Pecoraro, 2000).

Herein we report the synthesis, IR data, and the single-crystal X-ray structure of the title compound,C_28_H_16_Cu_5_N_4_O_12_, 2(C_8_H_20_N), 2(C_3_H_7_NO) abbreviated as (TEA)_2_[Cu(12—MC_Cu^II^N(shi)_-4](DMF)_2_, (1), where TEA is tetraethylammonium, shi^3-^ is salicylhydroximate, and DMF is *N,N*-dimethylformamide. The single-crystal X-ray structure of a related molecule, (TMA)_2_[Cu(12—MC_Cu^II^N(shi)_-4]^.^DMF (2, where TMA is tetramethylammonium), has previously been reported by Gibney *et al.* (1994), and the synthesis of another related molecule, (TEA)_2_[Cu(12—MC_Cu^II^N(d2shi)_-4)]^.^2DMF^.^H_2_O (where d_2_shi is 3,5-dideuteriosalicylhydroximate), has been described by Gibney *et al.* (1994).

Compound 1 is fairly planar, which is typical of Cu^II^ 12-MC-4 structures (Fig. 1–3; Macrae *et al.*, 2006). The structure consists of a [Cu^II^—N—O] repeat unit around the MC ring, and the MC binds a Cu^II^ in the central cavity. Cu1 is located in the central cavity and is four-coordinate with square planar geometry. Cu2, Cu3, Cu2^i^ and Cu3^i^ compose the MC ring (symmetry operator (i): -*x* + 1, -*y* + 1, -*z* + 1). Cu2 is five-coordinate with distorted square pyramidal geometry with τ equal to 0.02 (τ = 0 for square pyramidal geometry and τ = 1 for trigonal bipyramidal geometry (Addison *et al.*, 1984). The basal portion of the geometry is composed of two shi^3-^ ligands that bind with oxygen and nitrogen atoms. The apical position is filled by a DMF molecule which binds with an oxygen atom (O7 and O7b). The Cu2—O7 bond distance is 2.763 (14) Å, and the Cu2—O7b bond distance is 2.696 (17) Å. Cu3 is four-coordinate with square planar geometry, and the coordination is composed of two shi^3-^ ligands that bind with oxygen and nitrogen atoms. An uncoordinated TEA countercation is located in the lattice. In addition, the structure of 1 shows severe static disorder as the metallacrown, TEA, and DMF are disordered over two mutually exclusive sites (Figs. 4–6, Farrugia, 1997).

Compounds 1 and 2 are similar planar 12-MC-4 molecules. Compound 2 also consist of a [Cu^II^—N—O] repeat unit with a Cu^II^ ion bound in the central cavity (Gibney *et al.*, 1994). However, in 2 all of the ring Cu^II^ ions are four-coordinate with square planar geometry. The geometry about the ring Cu^II^ ions in 2 is different compared to 1. In 1 the DMF molecules are bound to two of the ring Cu^II^ ions, which gives these Cu^II^ ions a distorted square pyramidal geometry (Fig. 2). In 2 the DMF molecule does not bind to any of the Cu^II^ ions, but instead the DMF is present only in the lattice (Gibney *et al.*, 1994).

### Experimental

Copper(II) acetate monohydrate (99+%) was purchased from Sigma-Aldrich, salicylhydroxamic acid (H_3_shi, 99%) was purchased from Alfa Aesar, tetraethylammonium acetate (99%) was purchased from Acros Organics, absolute diethyl ether was purchased from EMD Chemicals, and *N,N*-dimethylformamide (ACS grade) was purchased from Fisher Scientific. All reagents were used as received and without further purification.

Copper(II) acetate monohydrate (0.625 mmol), salicylhydroxamic acid (0.5 mmol), and tetraethylammonium acetate (1.0 mmol) were mixed in 10 mL of DMF. Upon mixing the solution turned a dark green color. After stirring overnight, the solution was gravity filtered. No precipitate was observed, and the filtrate remained a dark green color. X-ray quality crystals were grown *via* diffusion of diethyl ether at 277 K (4 *^o^*C). The product was a dark green diamond-shaped crystal, and after washing the filtered product with cold DMF, the percent yield was 36% (0.0607 g) based on copper(II) acetate monohydrate. Elemental analysis for C_50_H_70_Cu_5_N_8_O_14_ [FW = 1325.74 g/mol] found % (calculated); C 45.21 (45.33); H 5.33 (5.33); N 8.37 (8.46).

### Refinement

The structure of 1 shows severe static disorder. The anionic metallacrown, the tetraethylammonium and the solvent DMF molecules all show disorder over each two mutually exclusive sites with different occupancy ratios. The refined values are 0.6215 (6) to 0.3785 (6) for the metallacrown, 0.759 (3) to 0.241 (1) for the tetraethylammonium ions and 0.537 (6) to 0.463 (6) for the DMF molecules. The metallacrown is disordered by a non-crystallographic two-fold axis, as is the DMF molecule. The tetraethylammonium is disordered by a pseudo-inversion center. Equivalent bonds in disordered sections of the molecules were restrained to be similar (standard deviation 0.02 Å). The atom O7 and O7b were restrained to be approximately isotropic (standard deviation 0.01 Å^2^), and the ADPs of the atoms C18*b* and C22, O7 and O7b, and N4 and N4b were each constrained to be the same. Aromatic benzene rings were constrained to resemble ideal hexagons with C—C distances of 1.39 Ångstroms.

Hydrogen atoms were placed in calculated positions with C—H = 0.95 (aromatic), 0.98 (methyl) and 0.99 Å (methylene) and were refined with Uĩso~(H) = 1.5 *U*_eq_(C) for methyl H atoms and 1.2 *U*_eq_(C) for methylene and aromatic moieties.

### Crystal data

**Table:**

(C_8_H_20_N)_2_[Cu_5_(C_7_H_4_NO_3_)_4_(C_3_H_7_NO)_2_]	*F*(000) = 2732
*M**_r_* = 1325.74	*D*_x_ = 1.672 Mg m^−^^3^
Orthorhombic, *P**b**c**a*	Mo *K*α radiation, λ = 0.71073 Å
Hall symbol: -P 2ac 2ab	Cell parameters from 9969 reflections
*a* = 16.641 (3) Å	θ = 2.4–28.2°
*b* = 13.616 (2) Å	µ = 2.06 mm^−^^1^
*c* = 23.238 (4) Å	*T* = 100 K
*V* = 5265.4 (15) Å^3^	Block, black
*Z* = 4	0.45 × 0.40 × 0.29 mm

### Data collection

**Table:**

Bruker SMART APEX CCD diffractometer	8316 independent reflections
Radiation source: fine-focus sealed tube	6387 reflections with *I* > 2σ(*I*)
graphite	*R*_int_ = 0.055
ω scans	θ_max_ = 31.4°, θ_min_ = 2.1°
Absorption correction: multi-scan (*APEX2*; Bruker, 2009)	*h* = −24→24
*T*_min_ = 0.588, *T*_max_ = 0.746	*k* = −19→19
51635 measured reflections	*l* = −31→33

### Refinement

**Table:**

Refinement on *F*^2^	Primary atom site location: structure-invariant direct methods
Least-squares matrix: full	Secondary atom site location: difference Fourier map
*R*[*F*^2^ > 2σ(*F*^2^)] = 0.043	Hydrogen site location: inferred from neighbouring sites
*wR*(*F*^2^) = 0.104	H-atom parameters constrained
*S* = 1.12	*w* = 1/[σ^2^(*F*_o_^2^) + (0.*P*)^2^ + 7.2598*P*] where *P* = (*F*_o_^2^ + 2*F*_c_^2^)/3
8316 reflections	(Δ/σ)_max_ = 0.002
631 parameters	Δρ_max_ = 0.47 e Å^−^^3^
101 restraints	Δρ_min_ = −0.35 e Å^−^^3^
3 constraints	

### Special details

**Table:**

Experimental. The structure of 1 shows severe static disorder. The anionic metallacrown, the tetraethylammonium, and the solvent DMF molecules all show disorder over each two mutually exclusive sites with different occupancy ratios. The refined values are 0.6215 (6) to 0.3785 (6) for the metallacrown, 0.759 (3) to 0.241 (1) for the tetraethylammonium ions and 0.537 (6) to 0.463 (6) for the DMF molecules. The metallacrown is disordered by a non-crystallographic two fold axis, as is the DMF molecule. The tetraethylammonium is disordered by a pseudo-inversion center. Equivalent bonds in disordered sections of the molecules were restrained to be similar (standard deviation 0.02 Å). The atom O7 and O7b were restrained to be approximately isotropic (standard deviation 0.01 Å^2^), and the ADPs of the atoms C18*b* and C22, O7 and O7b, and N4 and N4b were each constrained to be the same. Aromatic benzene rings were constrained to resemble ideal hexagons with C—C distances of 1.39 Å.IR bands (cm^-1^): 1605(*s*), 1572(*s*), 1526(*s*), 1437(*m*), 1389(*s*), 1319(*s*), 1254(*s*), 1097(*m*), 1024(*m*), 943(*m*), 742(*m*), 684(*m*), 657(*m*), 582(*m*), 476(*m*).
Geometry. All e.s.d.'s (except the e.s.d. in the dihedral angle between two l.s. planes) are estimated using the full covariance matrix. The cell e.s.d.'s are taken into account individually in the estimation of e.s.d.'s in distances, angles and torsion angles; correlations between e.s.d.'s in cell parameters are only used when they are defined by crystal symmetry. An approximate (isotropic) treatment of cell e.s.d.'s is used for estimating e.s.d.'s involving l.s. planes.
Refinement. Refinement of *F*^2^ against ALL reflections. The weighted *R*-factor *wR* and goodness of fit *S* are based on *F*^2^, conventional *R*-factors *R* are based on *F*, with *F* set to zero for negative *F*^2^. The threshold expression of *F*^2^ > σ(*F*^2^) is used only for calculating *R*-factors(gt) *etc*. and is not relevant to the choice of reflections for refinement. *R*-factors based on *F*^2^ are statistically about twice as large as those based on *F*, and *R*- factors based on ALL data will be even larger.

### Fractional atomic coordinates and isotropic or equivalent isotropic displacement parameters (Å^2^)

**Table:**

	*x*	*y*	*z*	*U*_iso_*/*U*_eq_	Occ. (<1)
Cu1	0.5000	0.5000	0.5000	0.02182 (9)	
Cu2	0.57625 (3)	0.41430 (3)	0.616568 (19)	0.02373 (11)	0.6215 (7)
O1	0.41463 (15)	0.57229 (17)	0.46572 (10)	0.0221 (5)	0.6215 (7)
N1	0.3913 (12)	0.6588 (11)	0.4928 (5)	0.0215 (16)	0.6215 (7)
C1	0.3635 (2)	0.7238 (3)	0.45496 (15)	0.0231 (7)	0.6215 (7)
O2	0.36367 (16)	0.7058 (2)	0.40009 (11)	0.0269 (5)	0.6215 (7)
C2	0.33280 (17)	0.81957 (17)	0.4762 (2)	0.0211 (8)	0.6215 (7)
C3	0.3010 (2)	0.8806 (3)	0.43404 (12)	0.0292 (10)	0.6215 (7)
H3	0.3017	0.8607	0.3949	0.035*	0.6215 (7)
C4	0.2680 (3)	0.9708 (3)	0.44919 (16)	0.0366 (14)	0.6215 (7)
H4	0.2463	1.0125	0.4204	0.044*	0.6215 (7)
C5	0.2669 (3)	0.9999 (2)	0.50651 (19)	0.0390 (15)	0.6215 (7)
H5	0.2444	1.0616	0.5169	0.047*	0.6215 (7)
C6	0.2988 (3)	0.9389 (3)	0.54869 (12)	0.0290 (9)	0.6215 (7)
H6	0.2980	0.9588	0.5879	0.035*	0.6215 (7)
C7	0.33169 (17)	0.8487 (2)	0.53355 (16)	0.0240 (8)	0.6215 (7)
O3	0.35984 (17)	0.7964 (2)	0.57879 (12)	0.0292 (6)	0.6215 (7)
Cu3	0.41466 (3)	0.67697 (3)	0.573321 (18)	0.02160 (11)	0.6215 (7)
O4	0.49116 (17)	0.57526 (19)	0.56729 (11)	0.0294 (6)	0.6215 (7)
N2	0.5163 (9)	0.5344 (12)	0.6212 (5)	0.024 (2)	0.6215 (7)
C8	0.4843 (2)	0.5834 (3)	0.66324 (15)	0.0240 (7)	0.6215 (7)
O5	0.43610 (16)	0.65689 (19)	0.65495 (11)	0.0265 (5)	0.6215 (7)
C9	0.5018 (2)	0.5490 (3)	0.72258 (11)	0.0232 (8)	0.6215 (7)
C10	0.4681 (2)	0.6052 (2)	0.7663 (2)	0.0293 (11)	0.6215 (7)
H10	0.4391	0.6633	0.7572	0.035*	0.6215 (7)
C11	0.4768 (3)	0.5765 (3)	0.82335 (16)	0.0412 (16)	0.6215 (7)
H11	0.4538	0.6149	0.8532	0.049*	0.6215 (7)
C12	0.5193 (3)	0.4915 (4)	0.83670 (10)	0.0396 (17)	0.6215 (7)
H12	0.5252	0.4719	0.8757	0.047*	0.6215 (7)
C13	0.5530 (2)	0.4353 (2)	0.7930 (2)	0.0305 (10)	0.6215 (7)
H13	0.5819	0.3773	0.8021	0.037*	0.6215 (7)
C14	0.54424 (19)	0.4641 (3)	0.73593 (15)	0.0265 (8)	0.6215 (7)
O6	0.58043 (19)	0.4037 (2)	0.69748 (12)	0.0336 (6)	0.6215 (7)
Cu2B	0.39897 (4)	0.64754 (5)	0.42099 (3)	0.02356 (18)	0.3785 (7)
O1B	0.5618 (2)	0.4844 (3)	0.56838 (16)	0.0223 (8)	0.3785 (7)
N1B	0.5321 (13)	0.5252 (18)	0.6190 (8)	0.022 (3)	0.3785 (7)
C1B	0.5508 (3)	0.4708 (4)	0.6645 (2)	0.0225 (11)	0.3785 (7)
O2B	0.5924 (3)	0.3906 (3)	0.6598 (2)	0.0304 (10)	0.3785 (7)
C2B	0.5244 (3)	0.5036 (5)	0.72271 (17)	0.0189 (12)	0.3785 (7)
C3B	0.5475 (4)	0.4426 (3)	0.7677 (3)	0.0265 (16)	0.3785 (7)
H3B	0.5775	0.3847	0.7600	0.032*	0.3785 (7)
C4B	0.5269 (6)	0.4664 (5)	0.8240 (2)	0.037 (3)	0.3785 (7)
H4B	0.5427	0.4247	0.8547	0.045*	0.3785 (7)
C5B	0.4830 (6)	0.5511 (6)	0.83522 (17)	0.038 (3)	0.3785 (7)
H5B	0.4689	0.5673	0.8737	0.045*	0.3785 (7)
C6B	0.4598 (4)	0.6121 (4)	0.7902 (3)	0.0236 (14)	0.3785 (7)
H6B	0.4299	0.6700	0.7979	0.028*	0.3785 (7)
C7B	0.4805 (3)	0.5883 (4)	0.7340 (2)	0.0212 (12)	0.3785 (7)
O3B	0.4532 (3)	0.6525 (3)	0.69378 (19)	0.0265 (9)	0.3785 (7)
Cu3B	0.45480 (4)	0.63044 (5)	0.61447 (3)	0.02077 (18)	0.3785 (7)
O4B	0.4357 (3)	0.5941 (3)	0.53712 (17)	0.0242 (8)	0.3785 (7)
N2B	0.394 (2)	0.6670 (17)	0.5039 (8)	0.021 (3)	0.3785 (7)
C8B	0.3775 (3)	0.7426 (4)	0.5357 (2)	0.0235 (11)	0.3785 (7)
O5B	0.3936 (3)	0.7458 (3)	0.58976 (17)	0.0247 (9)	0.3785 (7)
C9B	0.3388 (3)	0.8271 (3)	0.5066 (3)	0.0192 (12)	0.3785 (7)
C10B	0.3119 (4)	0.9002 (5)	0.54365 (16)	0.0270 (16)	0.3785 (7)
H10B	0.3188	0.8932	0.5840	0.032*	0.3785 (7)
C11B	0.2749 (5)	0.9836 (5)	0.5216 (3)	0.036 (2)	0.3785 (7)
H11B	0.2565	1.0336	0.5469	0.044*	0.3785 (7)
C12B	0.2647 (5)	0.9939 (4)	0.4625 (3)	0.032 (2)	0.3785 (7)
H12B	0.2394	1.0509	0.4474	0.039*	0.3785 (7)
C13B	0.2916 (4)	0.9207 (4)	0.42551 (17)	0.0266 (14)	0.3785 (7)
H13B	0.2847	0.9277	0.3851	0.032*	0.3785 (7)
C14B	0.3286 (3)	0.8373 (3)	0.4476 (3)	0.0206 (12)	0.3785 (7)
O6B	0.3489 (3)	0.7691 (3)	0.40768 (19)	0.0322 (10)	0.3785 (7)
N3	0.80044 (11)	0.22899 (15)	0.27205 (9)	0.0278 (4)	
C15	0.85084 (18)	0.2867 (2)	0.31451 (14)	0.0294 (7)	0.759 (3)
H15A	0.8953	0.2444	0.3282	0.035*	0.759 (3)
H15B	0.8751	0.3434	0.2942	0.035*	0.759 (3)
C16	0.8052 (4)	0.3239 (9)	0.3657 (3)	0.0357 (17)	0.759 (3)
H16A	0.7610	0.3660	0.3528	0.054*	0.759 (3)
H16B	0.8414	0.3619	0.3905	0.054*	0.759 (3)
H16C	0.7835	0.2682	0.3874	0.054*	0.759 (3)
C17	0.73347 (18)	0.2923 (2)	0.24704 (16)	0.0324 (8)	0.759 (3)
H17A	0.7047	0.2535	0.2175	0.039*	0.759 (3)
H17B	0.6946	0.3072	0.2781	0.039*	0.759 (3)
C18	0.7599 (3)	0.3870 (4)	0.2205 (2)	0.0417 (12)	0.759 (3)
H18A	0.7867	0.4275	0.2496	0.063*	0.759 (3)
H18B	0.7130	0.4222	0.2055	0.063*	0.759 (3)
H18C	0.7975	0.3735	0.1890	0.063*	0.759 (3)
C19	0.85842 (19)	0.1995 (3)	0.22333 (15)	0.0349 (8)	0.759 (3)
H19A	0.8831	0.2598	0.2074	0.042*	0.759 (3)
H19B	0.9021	0.1591	0.2399	0.042*	0.759 (3)
C20	0.8199 (4)	0.1429 (4)	0.1748 (3)	0.0452 (13)	0.759 (3)
H20A	0.7895	0.0875	0.1905	0.068*	0.759 (3)
H20B	0.8617	0.1183	0.1488	0.068*	0.759 (3)
H20C	0.7835	0.1863	0.1535	0.068*	0.759 (3)
C21	0.7619 (2)	0.1412 (2)	0.29905 (17)	0.0368 (8)	0.759 (3)
H21A	0.7276	0.1084	0.2700	0.044*	0.759 (3)
H21B	0.7266	0.1635	0.3307	0.044*	0.759 (3)
C22	0.8207 (6)	0.0671 (5)	0.3226 (3)	0.0457 (15)	0.759 (3)
H22A	0.8544	0.0421	0.2913	0.069*	0.759 (3)
H22B	0.7912	0.0126	0.3402	0.069*	0.759 (3)
H22C	0.8547	0.0986	0.3517	0.069*	0.759 (3)
C15B	0.7458 (6)	0.1682 (8)	0.2338 (4)	0.030 (2)	0.241 (3)
H15C	0.7221	0.1141	0.2566	0.035*	0.241 (3)
H15D	0.7014	0.2100	0.2195	0.035*	0.241 (3)
C16B	0.7909 (12)	0.1255 (15)	0.1830 (7)	0.042 (4)	0.241 (3)
H16D	0.8144	0.1789	0.1602	0.064*	0.241 (3)
H16E	0.7537	0.0877	0.1589	0.064*	0.241 (3)
H16F	0.8338	0.0823	0.1969	0.064*	0.241 (3)
C17B	0.8620 (6)	0.1601 (8)	0.3017 (5)	0.032 (2)	0.241 (3)
H17C	0.8985	0.1344	0.2717	0.038*	0.241 (3)
H17D	0.8948	0.2005	0.3282	0.038*	0.241 (3)
C18B	0.830 (2)	0.0743 (18)	0.3351 (12)	0.0457 (15)	0.241 (3)
H18D	0.7952	0.0979	0.3660	0.069*	0.241 (3)
H18E	0.8753	0.0376	0.3518	0.069*	0.241 (3)
H18F	0.7997	0.0312	0.3094	0.069*	0.241 (3)
C19B	0.7420 (6)	0.2669 (7)	0.3214 (4)	0.029 (2)	0.241 (3)
H19C	0.7018	0.3114	0.3040	0.035*	0.241 (3)
H19D	0.7126	0.2098	0.3374	0.035*	0.241 (3)
C20B	0.7826 (14)	0.320 (3)	0.3702 (11)	0.036 (5)	0.241 (3)
H20D	0.8125	0.2728	0.3938	0.055*	0.241 (3)
H20E	0.7421	0.3528	0.3939	0.055*	0.241 (3)
H20F	0.8199	0.3690	0.3546	0.055*	0.241 (3)
C21B	0.8441 (6)	0.3090 (8)	0.2448 (5)	0.036 (2)	0.241 (3)
H21C	0.8844	0.2807	0.2182	0.043*	0.241 (3)
H21D	0.8736	0.3457	0.2749	0.043*	0.241 (3)
C22B	0.7923 (9)	0.3799 (13)	0.2118 (8)	0.043 (4)	0.241 (3)
H22D	0.7521	0.3434	0.1896	0.064*	0.241 (3)
H22E	0.8259	0.4185	0.1856	0.064*	0.241 (3)
H22F	0.7650	0.4241	0.2388	0.064*	0.241 (3)
O7	0.4383 (10)	0.3329 (7)	0.5707 (5)	0.046 (2)	0.537 (7)
C23	0.4581 (3)	0.2485 (4)	0.5501 (2)	0.0402 (15)	0.537 (7)
H23	0.4947	0.2087	0.5711	0.048*	0.537 (7)
N4	0.4289 (7)	0.2151 (9)	0.5002 (5)	0.0320 (13)	0.537 (7)
C24	0.3656 (4)	0.2678 (5)	0.4701 (4)	0.0466 (16)	0.537 (7)
H24A	0.3179	0.2259	0.4672	0.070*	0.537 (7)
H24B	0.3842	0.2852	0.4314	0.070*	0.537 (7)
H24C	0.3522	0.3277	0.4914	0.070*	0.537 (7)
C25	0.4551 (4)	0.1234 (4)	0.4765 (4)	0.0528 (19)	0.537 (7)
H25A	0.4099	0.0772	0.4756	0.079*	0.537 (7)
H25B	0.4983	0.0963	0.5003	0.079*	0.537 (7)
H25C	0.4749	0.1339	0.4372	0.079*	0.537 (7)
O7B	0.4483 (11)	0.3087 (10)	0.5815 (6)	0.046 (2)	0.463 (7)
C23B	0.4126 (4)	0.3086 (5)	0.5331 (4)	0.052 (2)	0.463 (7)
H23B	0.3855	0.3664	0.5210	0.062*	0.463 (7)
N4B	0.4120 (9)	0.2298 (11)	0.4988 (6)	0.0320 (13)	0.463 (7)
C24B	0.4578 (4)	0.1433 (6)	0.5139 (4)	0.0451 (18)	0.463 (7)
H24D	0.4937	0.1260	0.4820	0.068*	0.463 (7)
H24E	0.4211	0.0886	0.5215	0.068*	0.463 (7)
H24F	0.4898	0.1568	0.5484	0.068*	0.463 (7)
C25B	0.3773 (6)	0.2329 (10)	0.4413 (4)	0.069 (3)	0.463 (7)
H25D	0.3255	0.1988	0.4414	0.104*	0.463 (7)
H25E	0.4138	0.2007	0.4141	0.104*	0.463 (7)
H25F	0.3693	0.3015	0.4297	0.104*	0.463 (7)

### Atomic displacement parameters (Å^2^)

**Table:**

	*U*^11^	*U*^22^	*U*^33^	*U*^12^	*U*^13^	*U*^23^
Cu1	0.02558 (18)	0.02169 (17)	0.01818 (17)	0.00622 (14)	−0.00229 (14)	−0.00452 (14)
Cu2	0.0274 (2)	0.0264 (2)	0.0174 (2)	0.00638 (17)	−0.00076 (17)	−0.00123 (16)
O1	0.0262 (12)	0.0207 (11)	0.0193 (12)	0.0034 (9)	0.0006 (10)	−0.0032 (9)
N1	0.024 (2)	0.020 (3)	0.021 (4)	0.003 (2)	0.005 (3)	−0.005 (3)
C1	0.0205 (15)	0.0266 (17)	0.0222 (17)	0.0000 (13)	−0.0007 (13)	0.0018 (13)
O2	0.0312 (13)	0.0285 (14)	0.0210 (13)	0.0084 (11)	−0.0037 (10)	0.0003 (11)
C2	0.0214 (17)	0.0212 (17)	0.021 (2)	0.0031 (13)	0.0021 (19)	−0.003 (2)
C3	0.027 (2)	0.030 (3)	0.030 (2)	0.008 (2)	−0.0025 (17)	0.0045 (19)
C4	0.035 (3)	0.034 (3)	0.041 (3)	0.008 (2)	0.002 (2)	0.014 (3)
C5	0.034 (3)	0.024 (2)	0.059 (4)	0.0123 (19)	0.004 (3)	−0.003 (3)
C6	0.033 (2)	0.024 (2)	0.030 (2)	0.008 (2)	0.0035 (18)	−0.002 (2)
C7	0.0216 (18)	0.022 (2)	0.029 (3)	0.0071 (15)	0.0063 (16)	−0.0052 (16)
O3	0.0387 (15)	0.0248 (13)	0.0243 (14)	0.0118 (12)	−0.0002 (11)	−0.0041 (11)
Cu3	0.0256 (2)	0.0206 (2)	0.0186 (2)	0.00376 (16)	0.00108 (16)	−0.00308 (16)
O4	0.0437 (16)	0.0300 (13)	0.0145 (12)	0.0152 (12)	−0.0006 (11)	−0.0010 (10)
N2	0.030 (6)	0.030 (2)	0.012 (2)	0.008 (3)	0.000 (2)	−0.0020 (19)
C8	0.0219 (16)	0.0274 (17)	0.0227 (17)	−0.0002 (13)	0.0011 (13)	−0.0028 (14)
O5	0.0326 (14)	0.0301 (13)	0.0168 (13)	0.0083 (11)	0.0012 (10)	−0.0073 (10)
C9	0.019 (2)	0.034 (3)	0.0162 (18)	0.0028 (16)	0.0008 (15)	−0.0052 (19)
C10	0.0193 (19)	0.040 (2)	0.028 (3)	−0.0006 (16)	0.004 (2)	−0.007 (2)
C11	0.027 (3)	0.066 (4)	0.031 (3)	−0.001 (3)	0.008 (2)	−0.018 (3)
C12	0.026 (3)	0.074 (5)	0.019 (2)	−0.006 (3)	−0.0004 (18)	0.003 (3)
C13	0.022 (2)	0.048 (3)	0.022 (2)	0.0043 (17)	−0.001 (2)	0.001 (2)
C14	0.0218 (19)	0.041 (3)	0.017 (2)	−0.0017 (18)	−0.0054 (16)	0.0034 (17)
O6	0.0455 (17)	0.0390 (16)	0.0162 (14)	0.0137 (13)	−0.0015 (12)	−0.0011 (11)
Cu2B	0.0292 (4)	0.0255 (3)	0.0160 (3)	0.0097 (3)	−0.0015 (3)	−0.0015 (3)
O1B	0.027 (2)	0.026 (2)	0.0142 (18)	0.0063 (16)	0.0035 (15)	−0.0003 (15)
N1B	0.018 (7)	0.031 (7)	0.017 (4)	−0.002 (4)	0.006 (3)	−0.009 (3)
C1B	0.022 (3)	0.028 (3)	0.018 (3)	0.002 (2)	−0.003 (2)	−0.003 (2)
O2B	0.038 (3)	0.030 (2)	0.023 (2)	0.0163 (19)	−0.0105 (19)	−0.0007 (18)
C2B	0.022 (3)	0.028 (4)	0.006 (3)	0.004 (3)	−0.002 (2)	0.003 (3)
C3B	0.025 (3)	0.032 (3)	0.023 (4)	0.008 (2)	−0.016 (4)	0.003 (4)
C4B	0.040 (5)	0.042 (5)	0.030 (5)	−0.004 (4)	−0.014 (4)	0.015 (5)
C5B	0.026 (5)	0.076 (7)	0.011 (3)	−0.014 (5)	0.005 (3)	−0.012 (4)
C6B	0.017 (3)	0.034 (3)	0.019 (4)	0.003 (2)	0.000 (3)	0.000 (3)
C7B	0.016 (3)	0.033 (4)	0.015 (4)	0.003 (2)	0.001 (2)	−0.009 (3)
O3B	0.033 (2)	0.025 (2)	0.022 (2)	0.0069 (17)	0.0005 (18)	−0.0056 (17)
Cu3B	0.0239 (3)	0.0214 (3)	0.0170 (3)	0.0056 (3)	−0.0010 (3)	−0.0019 (3)
O4B	0.032 (2)	0.0220 (19)	0.019 (2)	0.0105 (16)	−0.0009 (16)	0.0014 (16)
N2B	0.030 (5)	0.016 (5)	0.017 (6)	0.011 (4)	0.004 (5)	0.000 (4)
C8B	0.025 (3)	0.024 (3)	0.022 (3)	0.004 (2)	−0.001 (2)	0.004 (2)
O5B	0.033 (2)	0.022 (2)	0.019 (2)	0.0075 (18)	0.0000 (17)	−0.0046 (16)
C9B	0.024 (3)	0.016 (3)	0.018 (3)	0.009 (2)	0.004 (3)	−0.006 (3)
C10B	0.036 (4)	0.027 (4)	0.018 (3)	0.012 (3)	0.004 (3)	−0.010 (3)
C11B	0.047 (6)	0.032 (5)	0.030 (4)	0.005 (4)	0.006 (4)	−0.016 (4)
C12B	0.030 (4)	0.016 (3)	0.050 (6)	0.013 (3)	−0.007 (4)	0.002 (4)
C13B	0.032 (4)	0.023 (4)	0.025 (3)	0.008 (3)	0.001 (3)	−0.003 (3)
C14B	0.022 (3)	0.026 (3)	0.014 (3)	0.005 (2)	−0.003 (3)	0.002 (2)
O6B	0.047 (3)	0.028 (2)	0.021 (2)	0.015 (2)	−0.0022 (19)	−0.0002 (18)
N3	0.0189 (8)	0.0309 (10)	0.0337 (11)	0.0012 (7)	−0.0027 (8)	−0.0086 (8)
C15	0.0238 (14)	0.0309 (15)	0.0336 (17)	−0.0045 (12)	−0.0062 (12)	−0.0050 (13)
C16	0.037 (4)	0.036 (3)	0.034 (3)	0.003 (3)	−0.003 (3)	−0.008 (2)
C17	0.0210 (14)	0.0342 (16)	0.0420 (19)	0.0067 (12)	−0.0070 (13)	−0.0128 (14)
C18	0.043 (3)	0.040 (2)	0.042 (3)	0.013 (2)	−0.005 (2)	−0.0051 (18)
C19	0.0244 (15)	0.0441 (19)	0.0361 (19)	0.0099 (13)	0.0027 (13)	−0.0090 (15)
C20	0.053 (4)	0.044 (3)	0.038 (3)	0.007 (2)	0.000 (2)	−0.014 (2)
C21	0.0337 (17)	0.0285 (16)	0.048 (2)	−0.0082 (13)	0.0070 (15)	−0.0092 (15)
C22	0.056 (3)	0.0320 (18)	0.050 (4)	0.0023 (16)	−0.003 (3)	−0.002 (2)
C15B	0.020 (4)	0.035 (5)	0.034 (5)	0.003 (4)	−0.009 (4)	−0.006 (4)
C16B	0.058 (12)	0.048 (9)	0.021 (6)	−0.011 (8)	0.006 (7)	−0.008 (5)
C17B	0.024 (5)	0.037 (5)	0.035 (6)	0.011 (4)	−0.001 (4)	0.003 (4)
C18B	0.056 (3)	0.0320 (18)	0.050 (4)	0.0023 (16)	−0.003 (3)	−0.002 (2)
C19B	0.025 (4)	0.028 (5)	0.034 (5)	0.008 (4)	−0.001 (4)	−0.006 (4)
C20B	0.042 (13)	0.032 (7)	0.036 (8)	−0.014 (12)	0.007 (9)	−0.016 (6)
C21B	0.029 (5)	0.042 (6)	0.037 (6)	−0.006 (4)	−0.009 (4)	0.004 (5)
C22B	0.039 (9)	0.043 (8)	0.045 (9)	−0.003 (7)	−0.005 (8)	0.012 (7)
O7	0.040 (4)	0.053 (5)	0.046 (4)	−0.009 (4)	0.006 (3)	−0.013 (3)
C23	0.030 (2)	0.049 (3)	0.041 (3)	−0.012 (2)	0.009 (2)	0.007 (2)
N4	0.015 (5)	0.041 (4)	0.0400 (15)	0.004 (2)	0.006 (2)	−0.005 (2)
C24	0.033 (3)	0.051 (4)	0.055 (4)	−0.007 (2)	−0.010 (3)	−0.006 (3)
C25	0.037 (3)	0.042 (3)	0.079 (6)	0.000 (2)	0.010 (3)	−0.018 (3)
O7B	0.040 (4)	0.053 (5)	0.046 (4)	−0.009 (4)	0.006 (3)	−0.013 (3)
C23B	0.036 (4)	0.044 (4)	0.075 (6)	−0.009 (3)	0.026 (4)	0.005 (4)
N4B	0.015 (5)	0.041 (4)	0.0400 (15)	0.004 (2)	0.006 (2)	−0.005 (2)
C24B	0.035 (3)	0.045 (4)	0.056 (5)	−0.005 (3)	0.005 (3)	0.002 (3)
C25B	0.059 (5)	0.110 (9)	0.039 (5)	−0.014 (5)	−0.014 (4)	0.017 (5)

### Geometric parameters (Å, °)

**Table:**

Cu1—O4^i^	1.875 (2)	C12B—C13B	1.3900
Cu1—O4	1.875 (2)	C12B—H12B	0.9500
Cu1—O4B	1.879 (4)	C13B—C14B	1.3900
Cu1—O4B^i^	1.879 (4)	C13B—H13B	0.9500
Cu1—O1	1.903 (2)	C14B—O6B	1.355 (5)
Cu1—O1^i^	1.903 (2)	N3—C21B	1.454 (11)
Cu1—O1B	1.905 (4)	N3—C21	1.495 (4)
Cu1—O1B^i^	1.905 (4)	N3—C15	1.515 (3)
Cu2—O6	1.887 (3)	N3—C15B	1.517 (9)
Cu2—N2	1.919 (12)	N3—C17	1.524 (4)
Cu2—O1^i^	1.927 (2)	N3—C19	1.541 (4)
Cu2—O2^i^	1.956 (3)	N3—C17B	1.550 (10)
O1—N1	1.390 (12)	N3—C19B	1.590 (10)
O1—Cu2^i^	1.927 (2)	C15—C16	1.499 (7)
N1—C1	1.331 (14)	C15—H15A	0.9900
N1—Cu3	1.927 (11)	C15—H15B	0.9900
C1—O2	1.298 (4)	C16—H16A	0.9800
C1—C2	1.485 (4)	C16—H16B	0.9800
O2—Cu2^i^	1.956 (3)	C16—H16C	0.9800
C2—C3	1.3900	C17—C18	1.496 (6)
C2—C7	1.3900	C17—H17A	0.9900
C3—C4	1.3900	C17—H17B	0.9900
C3—H3	0.9500	C18—H18A	0.9800
C4—C5	1.3900	C18—H18B	0.9800
C4—H4	0.9500	C18—H18C	0.9800
C5—C6	1.3900	C19—C20	1.509 (6)
C5—H5	0.9500	C19—H19A	0.9900
C6—C7	1.3900	C19—H19B	0.9900
C6—H6	0.9500	C20—H20A	0.9800
C7—O3	1.354 (4)	C20—H20B	0.9800
O3—Cu3	1.868 (3)	C20—H20C	0.9800
Cu3—O4	1.886 (3)	C21—C22	1.508 (9)
Cu3—O5	1.949 (3)	C21—H21A	0.9900
O4—N2	1.432 (12)	C21—H21B	0.9900
N2—C8	1.298 (12)	C22—H22A	0.9800
C8—O5	1.297 (4)	C22—H22B	0.9800
C8—C9	1.485 (4)	C22—H22C	0.9800
C9—C10	1.3900	C15B—C16B	1.514 (13)
C9—C14	1.3900	C15B—H15C	0.9900
C10—C11	1.3900	C15B—H15D	0.9900
C10—H10	0.9500	C16B—H16D	0.9800
C11—C12	1.3900	C16B—H16E	0.9800
C11—H11	0.9500	C16B—H16F	0.9800
C12—C13	1.3900	C17B—C18B	1.499 (15)
C12—H12	0.9500	C17B—H17C	0.9900
C13—C14	1.3900	C17B—H17D	0.9900
C13—H13	0.9500	C18B—H18D	0.9800
C14—O6	1.355 (4)	C18B—H18E	0.9800
Cu2B—O6B	1.879 (4)	C18B—H18F	0.9800
Cu2B—O1B^i^	1.928 (4)	C19B—C20B	1.505 (14)
Cu2B—N2B	1.946 (19)	C19B—H19C	0.9900
Cu2B—O2B^i^	1.953 (5)	C19B—H19D	0.9900
O1B—N1B	1.391 (18)	C20B—H20D	0.9800
O1B—Cu2B^i^	1.928 (4)	C20B—H20E	0.9800
N1B—C1B	1.329 (19)	C20B—H20F	0.9800
N1B—Cu3B	1.928 (18)	C21B—C22B	1.505 (12)
C1B—O2B	1.297 (7)	C21B—H21C	0.9900
C1B—C2B	1.492 (6)	C21B—H21D	0.9900
O2B—Cu2B^i^	1.953 (5)	C22B—H22D	0.9800
C2B—C3B	1.3900	C22B—H22E	0.9800
C2B—C7B	1.3900	C22B—H22F	0.9800
C3B—C4B	1.3900	O7—C23	1.288 (11)
C3B—H3B	0.9500	C23—N4	1.337 (9)
C4B—C5B	1.3900	C23—H23	0.9500
C4B—H4B	0.9500	N4—C25	1.434 (9)
C5B—C6B	1.3900	N4—C24	1.454 (8)
C5B—H5B	0.9500	C24—H24A	0.9800
C6B—C7B	1.3900	C24—H24B	0.9800
C6B—H6B	0.9500	C24—H24C	0.9800
C7B—O3B	1.357 (5)	C25—H25A	0.9800
O3B—Cu3B	1.867 (4)	C25—H25B	0.9800
Cu3B—O4B	1.891 (4)	C25—H25C	0.9800
Cu3B—O5B	1.958 (4)	O7B—C23B	1.272 (13)
O4B—N2B	1.434 (19)	C23B—N4B	1.338 (11)
N2B—C8B	1.297 (18)	C23B—H23B	0.9500
C8B—O5B	1.286 (7)	N4B—C24B	1.446 (10)
C8B—C9B	1.481 (6)	N4B—C25B	1.455 (11)
C9B—C10B	1.3900	C24B—H24D	0.9800
C9B—C14B	1.3900	C24B—H24E	0.9800
C10B—C11B	1.3900	C24B—H24F	0.9800
C10B—H10B	0.9500	C25B—H25D	0.9800
C11B—C12B	1.3900	C25B—H25E	0.9800
C11B—H11B	0.9500	C25B—H25F	0.9800
			
O4^i^—Cu1—O4	179.997 (1)	O5B—C8B—C9B	120.7 (5)
O4^i^—Cu1—O4B	143.14 (14)	N2B—C8B—C9B	116.8 (9)
O4—Cu1—O4B	36.86 (14)	C8B—O5B—Cu3B	111.6 (3)
O4^i^—Cu1—O4B^i^	36.86 (14)	C10B—C9B—C14B	120.0
O4—Cu1—O4B^i^	143.14 (14)	C10B—C9B—C8B	114.5 (5)
O4B—Cu1—O4B^i^	179.998 (1)	C14B—C9B—C8B	125.5 (5)
O4^i^—Cu1—O1	89.55 (11)	C11B—C10B—C9B	120.0
O4—Cu1—O1	90.45 (11)	C11B—C10B—H10B	120.0
O4B—Cu1—O1	54.15 (14)	C9B—C10B—H10B	120.0
O4B^i^—Cu1—O1	125.84 (14)	C10B—C11B—C12B	120.0
O4^i^—Cu1—O1^i^	90.45 (11)	C10B—C11B—H11B	120.0
O4—Cu1—O1^i^	89.55 (11)	C12B—C11B—H11B	120.0
O4B—Cu1—O1^i^	125.84 (14)	C13B—C12B—C11B	120.0
O4B^i^—Cu1—O1^i^	54.16 (14)	C13B—C12B—H12B	120.0
O1—Cu1—O1^i^	179.998 (1)	C11B—C12B—H12B	120.0
O4^i^—Cu1—O1B	126.32 (14)	C12B—C13B—C14B	120.0
O4—Cu1—O1B	53.67 (14)	C12B—C13B—H13B	120.0
O4B—Cu1—O1B	90.03 (17)	C14B—C13B—H13B	120.0
O4B^i^—Cu1—O1B	89.97 (17)	O6B—C14B—C13B	114.7 (5)
O1—Cu1—O1B	144.09 (13)	O6B—C14B—C9B	125.2 (5)
O1^i^—Cu1—O1B	35.91 (13)	C13B—C14B—C9B	120.0
O4^i^—Cu1—O1B^i^	53.68 (14)	C14B—O6B—Cu2B	127.0 (4)
O4—Cu1—O1B^i^	126.33 (14)	C21B—N3—C21	174.9 (5)
O4B—Cu1—O1B^i^	89.97 (17)	C21B—N3—C15	67.5 (5)
O4B^i^—Cu1—O1B^i^	90.03 (17)	C21—N3—C15	112.3 (2)
O1—Cu1—O1B^i^	35.91 (13)	C21B—N3—C15B	116.9 (6)
O1^i^—Cu1—O1B^i^	144.09 (13)	C21—N3—C15B	63.4 (4)
O1B—Cu1—O1B^i^	179.999 (1)	C15—N3—C15B	175.2 (4)
O6—Cu2—N2	91.7 (3)	C21B—N3—C17	77.0 (4)
O6—Cu2—O1^i^	173.29 (12)	C21—N3—C17	107.4 (2)
N2—Cu2—O1^i^	90.9 (3)	C15—N3—C17	111.1 (2)
O6—Cu2—O2^i^	96.57 (11)	C15B—N3—C17	69.3 (4)
N2—Cu2—O2^i^	171.8 (3)	C21B—N3—C19	64.1 (5)
O1^i^—Cu2—O2^i^	80.94 (10)	C21—N3—C19	111.6 (2)
N1—O1—Cu1	117.2 (6)	C15—N3—C19	105.5 (2)
N1—O1—Cu2^i^	113.0 (6)	C15B—N3—C19	78.6 (4)
Cu1—O1—Cu2^i^	113.93 (12)	C17—N3—C19	109.0 (2)
C1—N1—O1	111.1 (8)	C21B—N3—C17B	108.5 (6)
C1—N1—Cu3	128.8 (8)	C21—N3—C17B	67.2 (4)
O1—N1—Cu3	119.5 (8)	C15—N3—C17B	70.0 (4)
O2—C1—N1	121.6 (6)	C15B—N3—C17B	109.1 (6)
O2—C1—C2	119.5 (3)	C17—N3—C17B	174.1 (4)
N1—C1—C2	118.9 (6)	C19—N3—C17B	75.9 (4)
C1—O2—Cu2^i^	110.7 (2)	C21B—N3—C19B	112.1 (6)
C3—C2—C7	120.0	C21—N3—C19B	72.2 (4)
C3—C2—C1	114.9 (3)	C15—N3—C19B	72.6 (4)
C7—C2—C1	125.0 (3)	C15B—N3—C19B	103.5 (5)
C4—C3—C2	120.0	C17—N3—C19B	69.2 (4)
C4—C3—H3	120.0	C19—N3—C19B	176.2 (4)
C2—C3—H3	120.0	C17B—N3—C19B	106.2 (6)
C3—C4—C5	120.0	C16—C15—N3	114.3 (3)
C3—C4—H4	120.0	C16—C15—H15A	108.7
C5—C4—H4	120.0	N3—C15—H15A	108.7
C6—C5—C4	120.0	C16—C15—H15B	108.7
C6—C5—H5	120.0	N3—C15—H15B	108.7
C4—C5—H5	120.0	H15A—C15—H15B	107.6
C7—C6—C5	120.0	C18—C17—N3	115.4 (3)
C7—C6—H6	120.0	C18—C17—H17A	108.4
C5—C6—H6	120.0	N3—C17—H17A	108.4
O3—C7—C6	113.9 (3)	C18—C17—H17B	108.4
O3—C7—C2	126.1 (3)	N3—C17—H17B	108.4
C6—C7—C2	120.0	H17A—C17—H17B	107.5
C7—O3—Cu3	125.0 (2)	C20—C19—N3	114.6 (3)
O3—Cu3—O4	166.74 (13)	C20—C19—H19A	108.6
O3—Cu3—N1	94.6 (4)	N3—C19—H19A	108.6
O4—Cu3—N1	88.3 (4)	C20—C19—H19B	108.6
O3—Cu3—O5	98.35 (11)	N3—C19—H19B	108.6
O4—Cu3—O5	81.13 (11)	H19A—C19—H19B	107.6
N1—Cu3—O5	164.5 (5)	N3—C21—C22	114.1 (4)
N2—O4—Cu1	119.6 (6)	N3—C21—H21A	108.7
N2—O4—Cu3	114.7 (5)	C22—C21—H21A	108.7
Cu1—O4—Cu3	121.07 (14)	N3—C21—H21B	108.7
C8—N2—O4	109.8 (8)	C22—C21—H21B	108.7
C8—N2—Cu2	133.9 (8)	H21A—C21—H21B	107.6
O4—N2—Cu2	115.7 (7)	C16B—C15B—N3	111.7 (10)
O5—C8—N2	122.6 (6)	C16B—C15B—H15C	109.3
O5—C8—C9	120.2 (3)	N3—C15B—H15C	109.3
N2—C8—C9	117.2 (6)	C16B—C15B—H15D	109.3
C8—O5—Cu3	111.5 (2)	N3—C15B—H15D	109.3
C10—C9—C14	120.0	H15C—C15B—H15D	107.9
C10—C9—C8	115.2 (4)	C15B—C16B—H16D	109.5
C14—C9—C8	124.7 (4)	C15B—C16B—H16E	109.5
C9—C10—C11	120.0	H16D—C16B—H16E	109.5
C9—C10—H10	120.0	C15B—C16B—H16F	109.5
C11—C10—H10	120.0	H16D—C16B—H16F	109.5
C10—C11—C12	120.0	H16E—C16B—H16F	109.5
C10—C11—H11	120.0	C18B—C17B—N3	118.0 (15)
C12—C11—H11	120.0	C18B—C17B—H17C	107.8
C11—C12—C13	120.0	N3—C17B—H17C	107.8
C11—C12—H12	120.0	C18B—C17B—H17D	107.8
C13—C12—H12	120.0	N3—C17B—H17D	107.8
C14—C13—C12	120.0	H17C—C17B—H17D	107.1
C14—C13—H13	120.0	C17B—C18B—H18D	109.5
C12—C13—H13	120.0	C17B—C18B—H18E	109.5
O6—C14—C13	114.3 (4)	H18D—C18B—H18E	109.5
O6—C14—C9	125.7 (4)	C17B—C18B—H18F	109.5
C13—C14—C9	120.0	H18D—C18B—H18F	109.5
C14—O6—Cu2	126.5 (3)	H18E—C18B—H18F	109.5
O6B—Cu2B—O1B^i^	173.0 (2)	C20B—C19B—N3	115.1 (11)
O6B—Cu2B—N2B	91.4 (6)	C20B—C19B—H19C	108.5
O1B^i^—Cu2B—N2B	90.8 (6)	N3—C19B—H19C	108.5
O6B—Cu2B—O2B^i^	96.25 (18)	C20B—C19B—H19D	108.5
O1B^i^—Cu2B—O2B^i^	81.39 (17)	N3—C19B—H19D	108.5
N2B—Cu2B—O2B^i^	172.1 (6)	H19C—C19B—H19D	107.5
N1B—O1B—Cu1	117.9 (8)	C19B—C20B—H20D	109.5
N1B—O1B—Cu2B^i^	112.6 (9)	C19B—C20B—H20E	109.5
Cu1—O1B—Cu2B^i^	113.2 (2)	H20D—C20B—H20E	109.5
C1B—N1B—O1B	111.5 (13)	C19B—C20B—H20F	109.5
C1B—N1B—Cu3B	127.9 (12)	H20D—C20B—H20F	109.5
O1B—N1B—Cu3B	119.2 (12)	H20E—C20B—H20F	109.5
O2B—C1B—N1B	121.9 (9)	N3—C21B—C22B	114.6 (10)
O2B—C1B—C2B	119.1 (5)	N3—C21B—H21C	108.6
N1B—C1B—C2B	119.0 (9)	C22B—C21B—H21C	108.6
C1B—O2B—Cu2B^i^	110.1 (4)	N3—C21B—H21D	108.6
C3B—C2B—C7B	120.0	C22B—C21B—H21D	108.6
C3B—C2B—C1B	115.0 (6)	H21C—C21B—H21D	107.6
C7B—C2B—C1B	125.0 (6)	C21B—C22B—H22D	109.5
C2B—C3B—C4B	120.0	C21B—C22B—H22E	109.5
C2B—C3B—H3B	120.0	H22D—C22B—H22E	109.5
C4B—C3B—H3B	120.0	C21B—C22B—H22F	109.5
C5B—C4B—C3B	120.0	H22D—C22B—H22F	109.5
C5B—C4B—H4B	120.0	H22E—C22B—H22F	109.5
C3B—C4B—H4B	120.0	O7—C23—N4	122.2 (8)
C4B—C5B—C6B	120.0	O7—C23—H23	118.9
C4B—C5B—H5B	120.0	N4—C23—H23	118.9
C6B—C5B—H5B	120.0	C23—N4—C25	121.3 (7)
C7B—C6B—C5B	120.0	C23—N4—C24	120.9 (7)
C7B—C6B—H6B	120.0	C25—N4—C24	117.7 (7)
C5B—C6B—H6B	120.0	O7B—C23B—N4B	122.1 (10)
O3B—C7B—C6B	114.5 (6)	O7B—C23B—H23B	118.9
O3B—C7B—C2B	125.5 (6)	N4B—C23B—H23B	118.9
C6B—C7B—C2B	120.0	C23B—N4B—C24B	120.3 (9)
C7B—O3B—Cu3B	124.8 (4)	C23B—N4B—C25B	121.8 (11)
O3B—Cu3B—O4B	167.9 (2)	C24B—N4B—C25B	117.1 (9)
O3B—Cu3B—N1B	94.3 (6)	N4B—C24B—H24D	109.5
O4B—Cu3B—N1B	88.2 (6)	N4B—C24B—H24E	109.5
O3B—Cu3B—O5B	98.81 (17)	H24D—C24B—H24E	109.5
O4B—Cu3B—O5B	80.99 (16)	N4B—C24B—H24F	109.5
N1B—Cu3B—O5B	163.5 (6)	H24D—C24B—H24F	109.5
N2B—O4B—Cu1	119.9 (9)	H24E—C24B—H24F	109.5
N2B—O4B—Cu3B	114.3 (7)	N4B—C25B—H25D	109.5
Cu1—O4B—Cu3B	121.3 (2)	N4B—C25B—H25E	109.5
C8B—N2B—O4B	110.2 (12)	H25D—C25B—H25E	109.5
C8B—N2B—Cu2B	132.9 (14)	N4B—C25B—H25F	109.5
O4B—N2B—Cu2B	114.8 (12)	H25D—C25B—H25F	109.5
O5B—C8B—N2B	122.6 (9)	H25E—C25B—H25F	109.5
			
O4^i^—Cu1—O1—N1	−166.7 (10)	N1B—C1B—C2B—C7B	0.2 (15)
O4—Cu1—O1—N1	13.3 (10)	C7B—C2B—C3B—C4B	0.0
O4B—Cu1—O1—N1	20.2 (10)	C1B—C2B—C3B—C4B	179.5 (5)
O4B^i^—Cu1—O1—N1	−159.8 (10)	C2B—C3B—C4B—C5B	0.0
O1B—Cu1—O1—N1	15.5 (10)	C3B—C4B—C5B—C6B	0.0
O1B^i^—Cu1—O1—N1	−164.5 (10)	C4B—C5B—C6B—C7B	0.0
O4^i^—Cu1—O1—Cu2^i^	−31.57 (14)	C5B—C6B—C7B—O3B	178.8 (5)
O4—Cu1—O1—Cu2^i^	148.43 (14)	C5B—C6B—C7B—C2B	0.0
O4B—Cu1—O1—Cu2^i^	155.3 (2)	C3B—C2B—C7B—O3B	−178.7 (5)
O4B^i^—Cu1—O1—Cu2^i^	−24.7 (2)	C1B—C2B—C7B—O3B	1.9 (6)
O1B—Cu1—O1—Cu2^i^	150.7 (2)	C3B—C2B—C7B—C6B	0.0
O1B^i^—Cu1—O1—Cu2^i^	−29.3 (2)	C1B—C2B—C7B—C6B	−179.4 (6)
Cu1—O1—N1—C1	149.6 (10)	C6B—C7B—O3B—Cu3B	−168.6 (4)
Cu2^i^—O1—N1—C1	14.0 (17)	C2B—C7B—O3B—Cu3B	10.1 (6)
Cu1—O1—N1—Cu3	−22.5 (17)	C7B—O3B—Cu3B—O4B	84.7 (9)
Cu2^i^—O1—N1—Cu3	−158.0 (9)	C7B—O3B—Cu3B—N1B	−17.2 (9)
O1—N1—C1—O2	−3(2)	C7B—O3B—Cu3B—O5B	172.8 (4)
Cu3—N1—C1—O2	167.9 (11)	C1B—N1B—Cu3B—O3B	20 (2)
O1—N1—C1—C2	177.8 (9)	O1B—N1B—Cu3B—O3B	−174.6 (17)
Cu3—N1—C1—C2	−11 (2)	O1B—N1B—Cu3B—O4B	17.3 (18)
N1—C1—O2—Cu2^i^	−8.9 (12)	C1B—N1B—Cu3B—O5B	163.0 (9)
C2—C1—O2—Cu2^i^	170.1 (2)	O1B—N1B—Cu3B—O5B	−32 (4)
O2—C1—C2—C3	4.3 (4)	O1B—Cu1—O4B—Cu3B	0.2 (3)
N1—C1—C2—C3	−176.7 (11)	O1B^i^—Cu1—O4B—Cu3B	−179.8 (3)
O2—C1—C2—C7	−178.0 (3)	O3B—Cu3B—O4B—N2B	93.2 (19)
N1—C1—C2—C7	1.1 (12)	N1B—Cu3B—O4B—N2B	−164.3 (19)
C7—C2—C3—C4	0.0	O5B—Cu3B—O4B—N2B	3.2 (17)
C1—C2—C3—C4	177.9 (3)	O3B—Cu3B—O4B—Cu1	−110.7 (8)
C2—C3—C4—C5	0.0	N1B—Cu3B—O4B—Cu1	−8.2 (8)
C3—C4—C5—C6	0.0	O5B—Cu3B—O4B—Cu1	159.3 (3)
C4—C5—C6—C7	0.0	Cu1—O4B—N2B—C8B	−157.7 (16)
C5—C6—C7—O3	−179.5 (3)	Cu3B—O4B—N2B—C8B	−1(3)
C5—C6—C7—C2	0.0	Cu1—O4B—N2B—Cu2B	8(3)
C3—C2—C7—O3	179.4 (3)	Cu3B—O4B—N2B—Cu2B	164.5 (12)
C1—C2—C7—O3	1.7 (4)	O6B—Cu2B—N2B—C8B	−12 (3)
C3—C2—C7—C6	0.0	O1B^i^—Cu2B—N2B—C8B	175 (3)
C1—C2—C7—C6	−177.7 (3)	O6B—Cu2B—N2B—O4B	−173 (2)
C6—C7—O3—Cu3	−175.1 (2)	O1B^i^—Cu2B—N2B—O4B	14 (2)
C2—C7—O3—Cu3	5.5 (4)	O4B—N2B—C8B—O5B	−3(3)
C7—O3—Cu3—O4	91.1 (6)	Cu2B—N2B—C8B—O5B	−165 (2)
C7—O3—Cu3—N1	−10.8 (7)	O4B—N2B—C8B—C9B	176.5 (15)
C7—O3—Cu3—O5	177.8 (3)	Cu2B—N2B—C8B—C9B	14 (4)
C1—N1—Cu3—O3	14.2 (18)	N2B—C8B—O5B—Cu3B	6(2)
O1—N1—Cu3—O3	−175.2 (14)	C9B—C8B—O5B—Cu3B	−173.9 (4)
C1—N1—Cu3—O4	−152.8 (18)	O3B—Cu3B—O5B—C8B	−172.4 (4)
O1—N1—Cu3—O4	17.7 (14)	O4B—Cu3B—O5B—C8B	−4.7 (4)
C1—N1—Cu3—O5	160.7 (7)	N1B—Cu3B—O5B—C8B	45 (3)
O1—N1—Cu3—O5	−29 (4)	O5B—C8B—C9B—C10B	−8.9 (7)
O4B—Cu1—O4—N2	144.6 (8)	N2B—C8B—C9B—C10B	171.4 (19)
O4B^i^—Cu1—O4—N2	−35.4 (8)	O5B—C8B—C9B—C14B	170.7 (4)
O1—Cu1—O4—N2	153.9 (8)	N2B—C8B—C9B—C14B	−9(2)
O1^i^—Cu1—O4—N2	−26.1 (8)	C14B—C9B—C10B—C11B	0.0
O1B—Cu1—O4—N2	−24.5 (8)	C8B—C9B—C10B—C11B	179.6 (5)
O1B^i^—Cu1—O4—N2	155.5 (8)	C9B—C10B—C11B—C12B	0.0
O4B—Cu1—O4—Cu3	−9.9 (2)	C10B—C11B—C12B—C13B	0.0
O4B^i^—Cu1—O4—Cu3	170.1 (2)	C11B—C12B—C13B—C14B	0.0
O1—Cu1—O4—Cu3	−0.56 (18)	C12B—C13B—C14B—O6B	177.0 (5)
O1^i^—Cu1—O4—Cu3	179.44 (18)	C12B—C13B—C14B—C9B	0.0
O1B—Cu1—O4—Cu3	−178.9 (3)	C10B—C9B—C14B—O6B	−176.7 (6)
O1B^i^—Cu1—O4—Cu3	1.1 (3)	C8B—C9B—C14B—O6B	3.7 (6)
O3—Cu3—O4—N2	94.1 (10)	C10B—C9B—C14B—C13B	0.0
N1—Cu3—O4—N2	−163.4 (11)	C8B—C9B—C14B—C13B	−179.6 (6)
O5—Cu3—O4—N2	5.3 (8)	C13B—C14B—O6B—Cu2B	−179.7 (4)
O3—Cu3—O4—Cu1	−110.3 (5)	C9B—C14B—O6B—Cu2B	−2.8 (7)
N1—Cu3—O4—Cu1	−7.7 (7)	N2B—Cu2B—O6B—C14B	4.8 (12)
O5—Cu3—O4—Cu1	160.95 (19)	O2B^i^—Cu2B—O6B—C14B	−176.9 (5)
Cu1—O4—N2—C8	−161.1 (8)	C21B—N3—C15—C16	126.6 (8)
Cu3—O4—N2—C8	−5.0 (15)	C21—N3—C15—C16	−58.8 (6)
Cu1—O4—N2—Cu2	11.6 (13)	C17—N3—C15—C16	61.4 (6)
Cu3—O4—N2—Cu2	167.6 (6)	C19—N3—C15—C16	179.4 (6)
O6—Cu2—N2—C8	−6.1 (16)	C17B—N3—C15—C16	−112.4 (7)
O1^i^—Cu2—N2—C8	−179.9 (16)	C19B—N3—C15—C16	2.8 (7)
O6—Cu2—N2—O4	−176.5 (10)	C21B—N3—C17—C18	−5.5 (6)
O1^i^—Cu2—N2—O4	9.7 (10)	C21—N3—C17—C18	177.0 (3)
O4—N2—C8—O5	1.0 (16)	C15—N3—C17—C18	53.9 (4)
Cu2—N2—C8—O5	−169.8 (10)	C15B—N3—C17—C18	−131.2 (5)
O4—N2—C8—C9	178.0 (7)	C19—N3—C17—C18	−61.9 (4)
Cu2—N2—C8—C9	7.2 (19)	C19B—N3—C17—C18	114.6 (5)
N2—C8—O5—Cu3	3.3 (10)	C21B—N3—C19—C20	−123.5 (6)
C9—C8—O5—Cu3	−173.6 (2)	C21—N3—C19—C20	59.4 (4)
O3—Cu3—O5—C8	−171.2 (2)	C15—N3—C19—C20	−178.4 (4)
O4—Cu3—O5—C8	−4.6 (2)	C15B—N3—C19—C20	4.2 (5)
N1—Cu3—O5—C8	43 (2)	C17—N3—C19—C20	−59.0 (4)
O5—C8—C9—C10	−4.6 (4)	C17B—N3—C19—C20	117.4 (6)
N2—C8—C9—C10	178.3 (9)	C15—N3—C21—C22	−61.4 (4)
O5—C8—C9—C14	171.6 (3)	C15B—N3—C21—C22	120.9 (6)
N2—C8—C9—C14	−5.5 (10)	C17—N3—C21—C22	176.2 (4)
C14—C9—C10—C11	0.0	C19—N3—C21—C22	56.8 (5)
C8—C9—C10—C11	176.4 (3)	C17B—N3—C21—C22	−6.4 (6)
C9—C10—C11—C12	0.0	C19B—N3—C21—C22	−123.3 (5)
C10—C11—C12—C13	0.0	C21B—N3—C15B—C16B	55.7 (13)
C11—C12—C13—C14	0.0	C21—N3—C15B—C16B	−118.6 (12)
C12—C13—C14—O6	179.4 (3)	C17—N3—C15B—C16B	118.3 (12)
C12—C13—C14—C9	0.0	C19—N3—C15B—C16B	2.8 (11)
C10—C9—C14—O6	−179.3 (4)	C17B—N3—C15B—C16B	−67.7 (12)
C8—C9—C14—O6	4.7 (4)	C19B—N3—C15B—C16B	179.5 (11)
C10—C9—C14—C13	0.0	C21B—N3—C17B—C18B	177.3 (16)
C8—C9—C14—C13	−176.0 (3)	C21—N3—C17B—C18B	−5.6 (15)
C13—C14—O6—Cu2	175.9 (2)	C15—N3—C17B—C18B	120.6 (16)
C9—C14—O6—Cu2	−4.7 (4)	C15B—N3—C17B—C18B	−54.4 (17)
N2—Cu2—O6—C14	4.2 (6)	C19—N3—C17B—C18B	−126.8 (16)
O2^i^—Cu2—O6—C14	−175.8 (3)	C19B—N3—C17B—C18B	56.6 (17)
O4B—Cu1—O1B—N1B	12.4 (13)	C21B—N3—C19B—C20B	−60 (2)
O4B^i^—Cu1—O1B—N1B	−167.6 (13)	C21—N3—C19B—C20B	117 (2)
O4B—Cu1—O1B—Cu2B^i^	146.8 (2)	C15—N3—C19B—C20B	−4.2 (19)
O4B^i^—Cu1—O1B—Cu2B^i^	−33.2 (2)	C15B—N3—C19B—C20B	173 (2)
Cu2B^i^—O1B—N1B—C1B	11 (2)	C17—N3—C19B—C20B	−126 (2)
Cu1—O1B—N1B—Cu3B	−22 (2)	C17B—N3—C19B—C20B	58 (2)
Cu2B^i^—O1B—N1B—Cu3B	−156.3 (12)	C15—N3—C21B—C22B	−124.4 (12)
O1B—N1B—C1B—O2B	1(2)	C15B—N3—C21B—C22B	53.7 (13)
Cu3B—N1B—C1B—O2B	166.8 (13)	C17—N3—C21B—C22B	−4.8 (11)
O1B—N1B—C1B—C2B	179.5 (11)	C19—N3—C21B—C22B	114.1 (12)
Cu3B—N1B—C1B—C2B	−14 (3)	C17B—N3—C21B—C22B	177.5 (11)
N1B—C1B—O2B—Cu2B^i^	−11.7 (15)	C19B—N3—C21B—C22B	−65.6 (12)
C2B—C1B—O2B—Cu2B^i^	169.3 (4)	O7—C23—N4—C25	176.5 (12)
O2B—C1B—C2B—C3B	−0.2 (7)	O7—C23—N4—C24	−7(2)
N1B—C1B—C2B—C3B	−179.2 (14)	O7B—C23B—N4B—C24B	−5(2)
O2B—C1B—C2B—C7B	179.2 (4)	O7B—C23B—N4B—C25B	−174.3 (15)

Symmetry codes: (i) −*x*+1, −*y*+1, −*z*+1.
